# A machine learning-based clinical tool for diagnosing myopathy using multi-cohort microarray expression profiles

**DOI:** 10.1186/s12967-020-02630-3

**Published:** 2020-11-30

**Authors:** Andrew Tran, Chris J. Walsh, Jane Batt, Claudia C. dos Santos, Pingzhao Hu

**Affiliations:** 1 Division of Biostatistics, Dalla Lana School of Public Health, University of Toronto, Toronto, ON Canada; 2 Keenan Research Center for Biomedical Science, St. Michael’s Hospital, Toronto, ON Canada; 3 Institute of Medical Sciences and Department of Medicine, University of Toronto, Toronto, ON Canada; 4 Interdepartmental Division of Critical Care, St. Michael’s Hospital, University of Toronto, 30 Bond Street, Room 4-008, Toronto, ON M5B 1WB Canada; 5 Department of Biochemistry and Medical Genetics, University of Manitoba, 745 Bannatyne Avenue, Winnipeg, MB R3E 0J9 Canada; 6 Research Institute in Oncology and Hematology, Winnipeg, MB Canada

**Keywords:** Muscle diseases, Machine learning, Microarray, Clinical tool, Biomarker

## Abstract

**Background:**

Myopathies are a heterogenous collection of disorders characterized by dysfunction of skeletal muscle. In practice, myopathies are frequently encountered by physicians and precise diagnosis remains a challenge in primary care. Molecular expression profiles show promise for disease diagnosis in various pathologies. We propose a novel machine learning-based clinical tool for predicting muscle disease subtypes using multi-cohort microarray expression data.

**Materials and methods:**

Muscle tissue samples originating from 1260 patients with muscle weakness. Data was curated from 42 independent cohorts with expression profiles in public microarray gene expression repositories, which represent a broad range of patient ages and peripheral muscles. Cohorts were categorized into five muscle disease subtypes: immobility, inflammatory myopathies, intensive care unit acquired weakness (ICUAW), congenital, and chronic systemic disease. The data contains expression data on 34,099 genes. Data augmentation techniques were used to address class imbalances in the muscle disease subtypes. Support vector machine (SVM) models were trained on two-thirds of the 1260 samples based on the top selected gene signature using analysis of variance (ANOVA). The model was validated in the remaining samples using area under the receiver operator curve (AUC). Gene enrichment analysis was used to identify enriched biological functions in the gene signature.

**Results:**

The AUC ranges from 0.611 to 0.649 in the observed imbalanced data. Overall, using the augmented data, chronic systemic disease was the best predicted class with AUC 0.872 (95% confidence interval (CI): 0.824–0.920). The least discriminated classes were ICUAW with AUC 0.777 (95% CI: 0.668–0.887) and immobility with AUC 0.789 (95% CI: 0.716–0.861). Disease-specific gene set enrichment results showed that the gene signature was enriched in biological processes including neural precursor cell proliferation for ICUAW and aerobic respiration for congenital (false discovery rate q-value < 0.001).

**Conclusion:**

Our results present a well-performing molecular classification tool with the selected gene markers for muscle disease classification. In practice, this tool addresses an important gap in the literature on myopathies and presents a potentially useful clinical tool for muscle disease subtype diagnosis.

## Introduction

Skeletal muscle disease, or myopathies, encompass a broad collection of disorders characterized by skeletal muscle dysfunction [1]. Myopathies can be hereditary or acquired in nature, and an exhaustive discussion of these disorders is made difficult by the vast heterogeneity of causes and presentation of muscle disease in clinical practice. Muscle diseases can be generally categorized under five categories: (i) immobility, (ii) inflammatory myopathy, (iii) Intensive care unit (ICU) acquired weakness (ICUAW), (iv) congenital muscle diseases, and (v) chronic systemic diseases. These categories were chosen based on existing categorizations of myopathies [1] and the distinct histo-pathological differences which allow these diseases to be distinguished from one another. Immobility-related myopathy is common in patients who are critically ill and is caused by prolonged periods of bedrest [2]. ICUAW presents in similar patient demographics, and is defined as clinically detectable weakness which has no discernable causes other than critical illness. Inflammatory myopathy features chronic muscle inflammation and weakness without a known cause, and are considered to be rare diseases with low prevalence [3]. Congenital myopathy is caused by the failure of structural muscle proteins due to genetic defects, and onset usually occurs during the neonatal period [4]. Chronic systemic diseases, such as rheumatoid arthritis and systemic lupus erythematosus, are caused by maladaptation of various body systems including the immune, nervous, and endocrine systems [5]. Systemic disease is debilitating and often leads to muscle wasting due to increases in catabolic reactions which stem from dysregulated cell signalling [6]. These categories are broad and contain significant heterogeneity among individual diseases within each category.

In recent years, an increasing number of studies have reported potential underlying genetic mechanisms for myopathies. Gene expression profiling has demonstrated great promise as a clinical diagnostic tool particularly in oncology [7, 8]. However, there is a currently a lack of clinically relevant diagnostic biomarkers or molecular classification tools for muscle disease [9]. The abundance of expression profiling data on the public repositories Gene Expression Omnibus (GEO) and ArrayExpress presents a novel space for machine learning and artificial intelligence researchers to apply classification techniques towards muscle disease.

Studies which have identified abnormal gene expression profiles in patients with myopathy tend to be limited by small sample sizes and homogeneity that is unrepresentative of the real-world population [9]. Multi-cohort frameworks circumvent these issues by analyzing independent, heterogenous datasets. This type of analysis has identified gene signatures in other pathologies including sepsis and systemic sclerosis [10, 11].

We hypothesized that different muscle diseases have distinct molecular gene expression profiles which can be used for classification into five categories of muscle disease. These gene expression profiles can further improve our current understanding of the various biological mechanisms involved in different types of muscle disease. The primary objective was to build a molecular classification tool from multi-cohort microarray expression profile data using machine learning algorithms across five muscle disease categories: immobility, inflammatory, ICU acquired weakness, congenital, and chronic systemic disease. The secondary objective was to report potential clinical biomarkers from the gene signature identified by our classifier.

## Methods

**Fig. 1:**
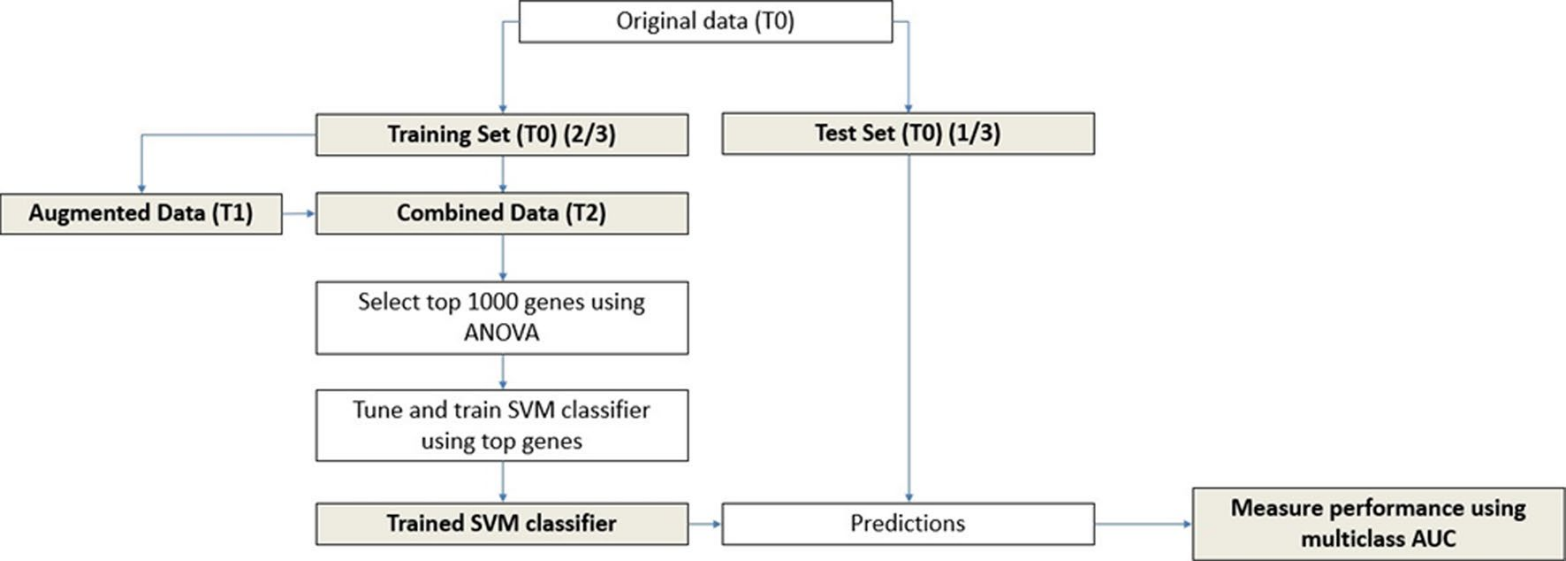
Model training and validation workflow. The original, augmented, and combined expression profile data are referred to as T0, T1, and T2 respectively. A training-test split of 2:1 was made for T0. The training set T2 was used for feature selection and training the support vector machine (SVM) classifier. The test set of T0 were used for making predictions and validating the model performance measured by multiclass area under the receiver-operator curve (AUC). This workflow was applied to three data augmentation strategies: (**a**) no class size adjustment, (**b**) sampling to the mean class size, and (**c**) sampling to twice the mean class size

The overall data analysis procedure is detailed in Fig. 1.

### Data collection

We collected microarray data which contains gene expression levels in patients with various forms of muscle disease from various cohorts. The data used in this study was collected and pre-processed in a previous study by our group [9]. Two major public microarray gene expression repositories (ArrayExpress from European Bioinformatics Institute and Gene Expression Omnibus from National Institute of Health) (search date: Aug 29, 2018) were searched for human muscle disease datasets. Datasets that met the following criteria were included in the study: (1) samples originated from human peripheral muscle tissue, (2) data was acquired using microarray platforms detecting > 10,000 genes, (3) the probe-to-gene mapping annotations were clear, (4) there were 5 >  = cases and 5 >  = controls in each dataset, and (5) the controls were derived from healthy muscle tissue. Samples taken after intervention (e.g. after cancer resection) were excluded. The datasets were classified into 5 muscle disease categories: immobility, inflammatory myopathies, ICU acquired weakness (ICUAW), congenital, and chronic systemic disease. All analyses carried out in this study were performed using R Version 3.6.3.

### Data pre-processing and data quality check

Using standardized methods, all microarray data was renormalized from raw data [9]. Affymetrix arrays were normalized using R package *affy* and non-Affymetrix arrays were normalized using R package *limma*. Probe-to-gene mappings were derived from SOFT files in GEO. All expression data was log-2 transformed because gene expression data is often heavily right-skewed in the linear scale [12]. A total of 34,099 genes were measured in at least one cohort and 2782 genes were measured in all cohorts. Only the common 2782 genes were considered for subsequent analyses, and all other genes can be considered “missing” for at least one cohort. To our knowledge, there are no guidelines for handling missing data in multi-cohort studies. However, guidelines for randomized clinical trials recommend skipping imputation and using only observed data when more than 40% of the data is missing [13]. Batch correction was performed to account for study-specific batch effects (see Additional file 1).

### Data augmentation

Two issues encountered with this dataset were class imbalances and low sample size. We employed a sampling strategy consisting of under- and over-sampling to address these issues because class imbalances and low sample sizes can lead to poor classification results [14]. Under-sampling was performed by randomly selecting a proportion of the samples from larger classes. Over-sampling is a form of data augmentation, which is a strategy that allows users to increase the amount and diversity of training data without collecting new observations [15]. Over-sampling was performed using Synthetic Minority Oversampling Technique (SMOTE). This over-sampling method was used to create augmented samples of the smaller classes which share feature similarities with existing samples (R package *UBL*). Complete documentation of the SMOTE algorithm has been described elsewhere [16].

Three data augmentation strategies were considered to generate balanced datasets: (**a**) no class size adjustment, (**b**) sampling to the mean class size, and (**c**) sampling to twice the mean class size. Training datasets generated by strategy (**b**) and (**c**) contain samples from the *original dataset* (T0) and *augmented samples* (T1). These datasets are referred to as *combined data* (T2).

### Feature selection

Since we have many genes and relatively few samples, we cannot build the classification model from the data directly, which will result in overfitting. Feature selection is performed to reduce the number of genes to improve interpretability of the classifier, reduce noise from irrelevant variables, and prevent overfitting [17, 18]. The ideal classifier performs well with a selected subset of genes. Important genes from the common set of 2782 genes were selected using one-way ANOVA in the training set of the original data before any adjustments were made to class size. ANOVA is used to select important genes because it can identify which genes are differentially expressed among the six groups. P-values were adjusted using the Benjamini–Hochberg method to account for multiple testing. The genes were ranked by adjusted p-value and the top 1000 genes were selected as features in the subsequent classification analysis.

### Training-test partition

The datasets were partitioned into training-test sets using a 2:1 split stratified by class. Data augmentation was performed only in the training set to reduce the chance of overfitting. The training-test partition was performed 30 times and model performance was averaged over all iterations to obtain stable performance measurements.

### Model training and validation

The support vector machine (SVM) model was trained using the training set from the combined data (T2) for both class size adjustment cases, respectively. The trained SVM models used a radial basis function and the cost parameter was tuned by tenfold cross validation in the training set. For the cases where class size adjustments were made, we measured performance in the original (T0) test set. The test set was used to predict class membership probabilities, and performance was measured using multiclass area under the receiver-operator curve (AUC).

Performance was measured as a function of the number of genes included in the SVM model. The AUC was calculated by averaging the results over the 30 training-test partitions. The gene set that generated an SVM model with stable AUC measurements was considered for further ROC curve and gene set enrichment analysis.

### Gene set enrichment analysis

The gene list used in our final classification model is generally named as the gene signature or gene biomarker. We evaluate the enrichment of relevant gene ontologies in our gene signature using gene set enrichment analysis (GSEA). GSEA uses Fisher’s exact test based on a hypergeometric distribution to determine whether known biological functions or pathways are over-represented in a given gene list [19]. Significant pathways in our 500-gene signature were determined using a false discovery rate (FDR) q-value threshold of 0.05 (Q < 0.05) and nominal p-value of 0.05 (p < 0.05). The importance scores from the binary disease-specific SVM models were used to rank the genes in our gene signature. Gene sets were searched in the Gene Ontology (GO) library using GSEA software using the pre-ranked list and results were visualized in an enrichment map with Cytoscape version 3.8.0.

### Availability of supporting data

The datasets used in this paper are publicly available in GEO (https://www.ebi.ac.uk/) and Array Express (https://www.ncbi.nlm.nih.gov/geo/). The accession numbers are listed in a previous paper [9].

## Results

### Sample characteristics

Our dataset is comprised of 42 independent cohorts containing 1293 samples (824 cases and 469 controls). After 33 duplicate control samples are removed, there are 1260 samples remaining (824 cases and 436 controls). The number of controls and cases, gene count, and microarray platform for each cohort can be found in Additional file 2. The cohorts represent a heterogenous population of patients with various types of muscle disease. The samples were categorized as control (N = 469), congenital (N = 386), inflammatory myositis (N = 123), immobile (N = 121), ICUAW (N = 49), or chronic (N = 145).

### Data augmentation

There appeared to be noteworthy class imbalance in our dataset, which motivates the use of data augmentation strategies. Class size adjustments were made using three data augmentation strategies: (**a**) no class size adjustment, (**b**) sampling to the mean class size, and (**c**) sampling to twice the mean class size. Each augmentation strategy was used to train and validate an SVM classifier. The model building procedure, as described in the methods, is displayed in Fig. 1.

### Built a gene signature-based multiclass classifier

**Fig. 2:**
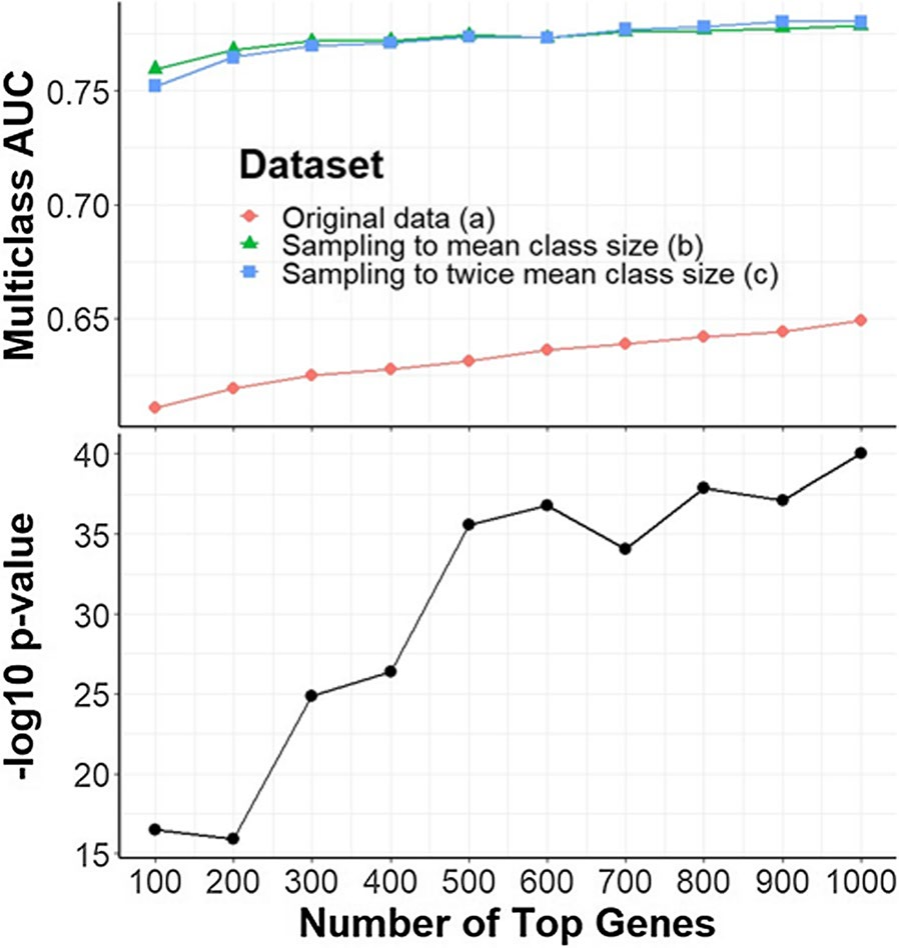
SVM model performance as a function of top genes using an augmentation strategy of no data augmentation (**a**) (N = 1260), sampling to the mean class size (**b**) (N = 1260), or sampling to twice the mean class size (**c**) (N = 2520). Model performance was averaged over 30 iterations. In strategy (**a**), the model was trained using the training set of the original data T0 with a 2:1 training-test split stratified by class. In strategies (**b**) and (**c**), the model was trained using the augmented training set T2 with a 2:1 training-test split stratified by class. Performance was measured by multiclass AUC in the test set of T0. The p-values in the bottom panel indicate the enrichment of the gene signature in muscle specific genes

Figure 2 (Top panel) displays the results of using data augmentation strategies (**a–c**) to train an SVM classifier with varying number of top genes. For strategy (**a**), the SVM classifier was trained and validated using only the original dataset. There appears to be a positive, linear association between gene number and performance, although the performance in general is relatively poor. The AUC ranges from 0.611 to 0.649 for this model. In strategies (**b–c**), the classifier was trained on balanced, augmented class data (N = 1260 and N = 2520 for strategy (**b**) and (**c**) respectively). Overall, the performance of both augmented classifiers was better and more stable than the performance of classifier (**a**). The AUC ranges from 0.760 to 0.779 and 0.752 to 0.780 using strategies (**b**) and (**c**) respectively. It is interesting to note that further inflating the sample size to twice the mean class size (**c**) after already balancing the classes (**b**) did not appear to appreciably improve classifier performance. For both augmentation strategies, the AUC appears to increase until approximately 500 genes, and then plateaus with additional genes. Additionally, a Fisher’s exact test was conducted to determine whether the gene signature was enriched with muscle specific genes. A similar plateau at approximately 500 genes is observed in the enrichment significance level measured by − log 10 p-values as shown in Fig. 2 (Bottom panel). It should be noted that there was significant enrichment in all of the cut-off thresholds up to 1000 genes. This is likely because we only analyzed the genes common among all the included studies. Since all of the studies are related to muscle disease, there is a greater likelihood that the overlap between these studies consist of muscle-related genes.

As a result, this is used as the cut-off for obtaining the gene signature used in the subsequent analyses. This cut-off was selected because it appeared to provide good classifier performance without including an excessive number of genes. The full list of genes, along with associated p-values and muscle specificity, is available as supplemental material (Additional file 3).

### Disease-specific classifier

**Fig. 3:**
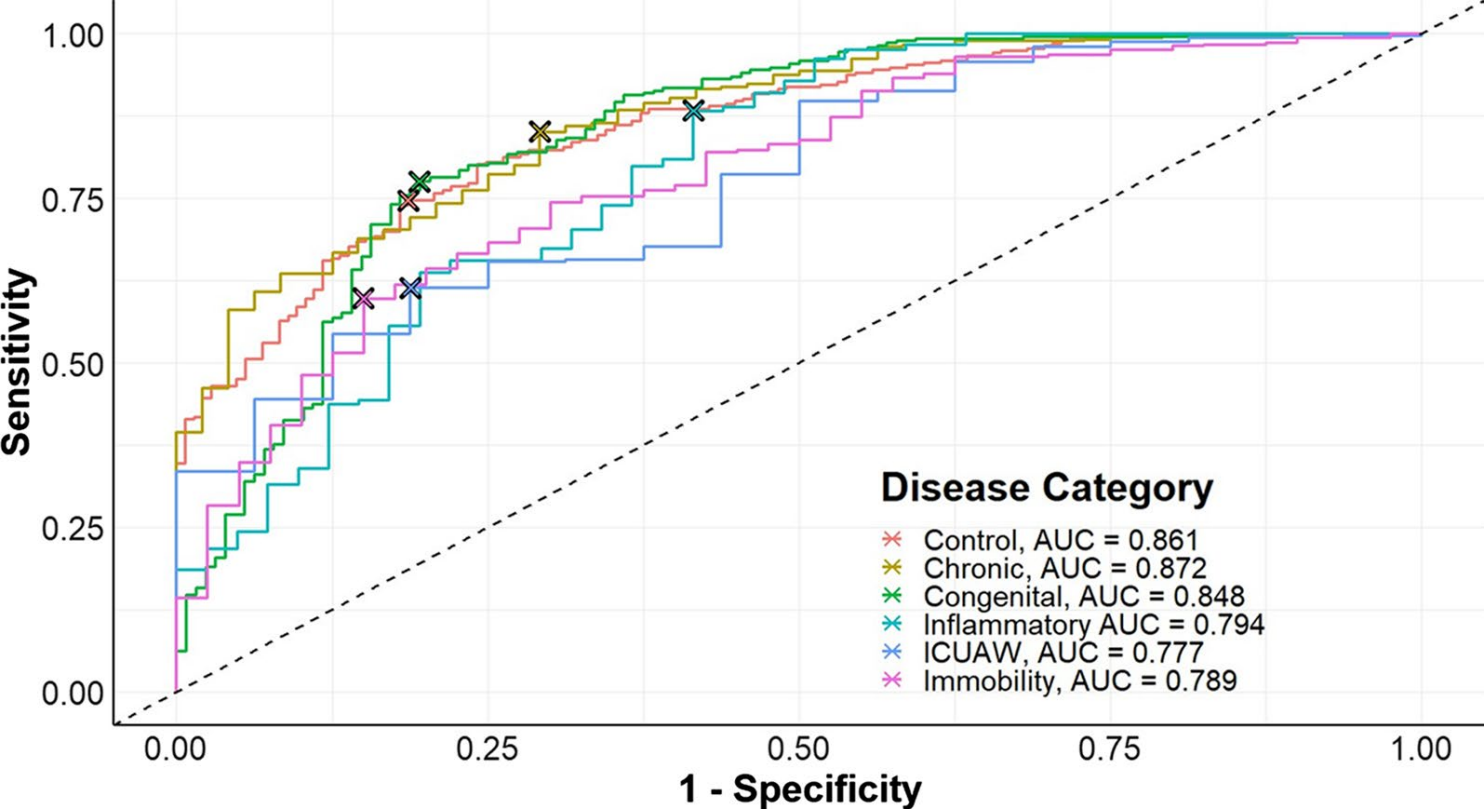
Disease-specific ROC curves for model discrimination using the top 500 genes. Classes were balanced using an augmentation strategy of sampling to twice the mean class size (N = 2520). Optimal thresholds were determined using a Youden’s J statistic and are indicated on each disease-specific ROC curve by crosses

As a follow-up to the multiclass analysis, we trained an SVM classifier and dichotomized the classification predictions into those belonging one specific class or any other class. Disease-specific ROC curves generated from the classifier trained on class-balanced data is shown in Fig. 3. The 95% bootstrapping confidence intervals for the class-specific AUC values are shown in Table 1. The specificity, sensitivity, precision, and F1-score were calculated at the optimal threshold for each disease-specific classification. Optimal thresholds were determined using the Youden’s J statistic. The classes were augmented to twice the mean class size and the model was validated using the original dataset. This classifier was trained using the same 500 genes identified in the previous section. Overall, the performance of the classifier appears to be relatively good for all classes. This classifier has good discrimination and appears to perform the best for the chronic systemic disease with AUC 0.872 (95% confidence interval (CI): 0.824–0.920). The least discriminated classes were ICUAW with AUC 0.777 (95% CI: 0.668–0.887) and immobility with AUC 0.789 (95% CI: 0.716–0.861).

**Table 1:** Disease-specific 95% confidence intervals for the AUC using the top 500 genes

Diseases	AUC (95% CI)	Specificity	Sensitivity	Precision	F1 score
Control	0.861 (0.826–0.895)	0.814	0.747	0.883	0.810
Chronic	0.872 (0.824–0.920)	0.708	0.851	0.957	0.901
Congenital	0.848 (0.805–0.892)	0.805	0.776	0.900	0.833
IM	0.794 (0.713–0.876)	0.585	0.883	0.951	0.916
ICUAW	0.777 (0.668–0.887)	0.812	0.614	0.988	0.758
Immobility	0.789 (0.716–0.861)	0.850	0.598	0.974	0.741

*IM* inflammatory myositis, *ICUAW* intensive care unit acquired weakness, *CI* confidence interval. Classes were balanced using an augmentation strategy of sampling to twice the mean class size (N = 2520). Confidence intervals were generated using 2000 stratified bootstrapping replications. Optimal thresholds were determined using a Youden’s J statistic. Specificity, sensitivity, precision, and F1 score were calculated at the optimal threshold

### Gene set enrichment analysis

**Fig. 4:**
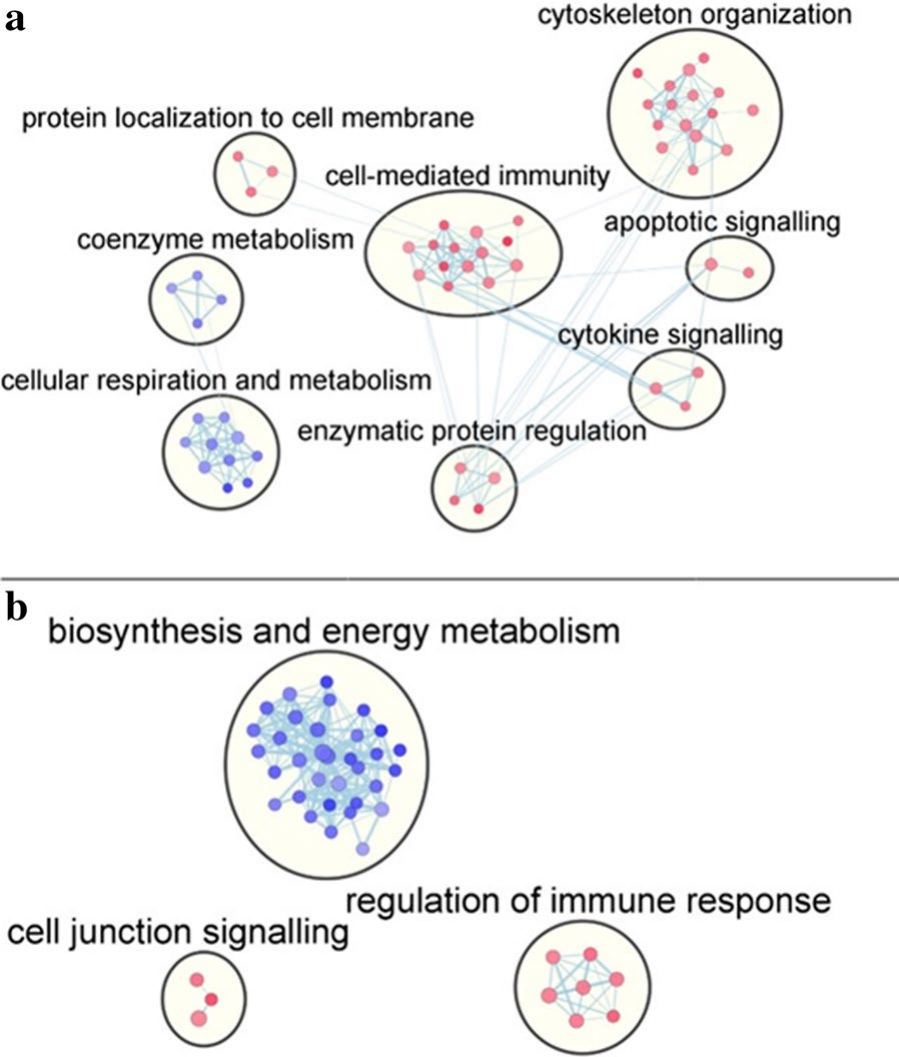
Enrichment map of the biological processes related to the top 500 genes identified by ANOVA included in the **a** congenital and **b** immobility disease classifier. Circles are referred to as “nodes” and the connectors are “edges”. Nodes represent specific biological pathways and node size represents the number of genes in the pathway. Edges connecting adjacent nodes represent overlapping pathways and edge width represents gene overlap size. The node colour represents enrichment score. Nodes that are blue are upregulated (enrichment score greater than zero) and nodes that are red are downregulated (enrichment score less than zero). The nodes are clustered into general functional groups

Gene set enrichment analysis of the top 500 genes identified from the multiclass analysis was performed using GSEA software to identify the biological processes that are enriched in our gene signature. These 500 genes are common to all the disease classifiers, but each gene was assigned a different importance score by their respective SVM classifier. These importance scores were used to generate a ranked list of genes for each disease for GSEA analysis. GSEA analysis was carried out for each muscle disease subtype using the pre-ranked genes. We report the top 5 disease-specific upregulated and downregulated biological pathways (FDR q-value < 0.05) in Additional file 4 and Additional file 5, respectively. Non-significant results were filtered from the table. The congenital and immobility muscle disease classes had the greatest number of enriched Gene Ontology Biological Process (GO BP) terms, and were visualized using an enrichment map in Fig. 4 using Cytoscape. Disease nonspecific GSEA analysis was also conducted (see Additional file 6).

## Discussion

We collected microarray expression profile data from 42 independent cohorts comprising of 1260 samples to build a molecular classification tool for five categories of muscle disease. To our present knowledge, there are currently no molecular classification tools for muscle disease. Thus, our research represents a unique and novel contribution to the muscle disease classification literature.

Before making any class adjustments to the data, the performance of the classifier was mediocre with an AUC ranging from 0.611 to 0.649. However, after class adjustments were made to balance the class distribution while keeping the total sample size the same, the AUC rose to above 0.75 in the test set of the original data. Features in minority classes are often treated as noise by machine learning algorithms and thus misclassification error tends to be greater for minority classes [8, 14]. This problem was remedied by sampling all classes to equal sizes and building the multiclass classifier from this combined, balanced data. The performance of the binary one vs. all disease classifiers was also excellent for all disease classes. The choice to generate multiple binary classifiers was motivated by practicality. In practice, binary classification tools are more interpretable and likely to be useful for differential diagnosis than multiclass classifiers.

Machine learning models for disease classification are seeing more use with the growth of publicly available clinical data. Some remarkable models have been used to classify 11 different types of neuromuscular disease with 100% accuracy using microarray data [20], and automate diagnosis of myositis from ultrasound images with up to 87% accuracy [21]. However, although machine learning models excel at making predictions from data, these methods fail to inform us about the biological mechanisms which differentiate one disease from another. This highlighted the need for additional methods which could provide biological insights, such as GSEA in this study.

From the non-specific GSEA analysis, we identified clustered biological processes such as actin cytoskeleton organization. These should appear unsurprising, as α-actin is a principal component of skeletal muscle, and many muscle diseases have been identified in relation to mutations of the α-actin gene *ACTA1*. However, non-specific analyses have limited utility in that they are broad and fail to reveal insights about disease-specific mechanisms. This is the rationale behind conducting separate GSEA analysis for each disease, which revealed disease-specific biological processes in our 500-gene signature. This analysis allowed us to make more contextual and focussed conclusions about the biology of each disease. ICUAW is a type of muscle weakness which occurs while patients are being treated for life-threatening, primary disorders. It is commonly classified into three components: critical illness polyneuropathy (CIP), critical illness myopathy (CIM), and critical illness neuromyopathy (CINM) [22]. The prevailing hypothesis in the pathophysiology of CIP is peripheral nervous system failure. During the catabolic state of critical illness, hypoperfusion from microcirculatory changes can contribute to neuronal injury and axon degeneration. From our GSEA results, one pathway which appears to be unique to ICUAW is neural precursor cell proliferation. Neural precursor cells comprise of stem cells and progenitor cells which expand and replace the neural cell population [23]. They can be found in the central nervous system and have been previously shown to play an important role in repairing damage in the brain following stroke [24]. However, the role of neurogenesis in the peripheral nervous system (PNS) is much less poorly understood. There is currently a lack of direct evidence regarding neurogenesis after insult to the PNS [25]. Other researchers have stipulated that stem cells can be engineered to modify their proliferation and differentiation to assist with PNS repair, but these claims require additional experimentation. Enrichment of neural precursor cell proliferation in the ICUAW cohort from our analysis indicates that endogenous activation of neural precursor cells may be occurring in patients afflicted with ICUAW. This is novel information which provides insight into the physiological responses to ICUAW and warrants further study into the of neural stem cells as a potential therapeutic target.

Another result of interest is the enrichment of metabolic processes such cellular respiration and oxidative energy derivation in the congenital and immobility categories of muscle disease. Metabolic myopathies are a group of rare and etiologically diverse disorders which are caused by defects in bioenergetic metabolism [26]. Due to the high energy requirement of skeletal muscle, insufficient metabolism results in progressive muscle wasting and persistent weakness. Defects may occur in any metabolic pathway, including glycogenolysis, Krebs cycle, or mitochondrial respiration. This class of muscle disease is predominantly caused by genetic defects that affect enzymatic functions. Although metabolic myopathy belongs to its own distinct classification, our analysis suggests that there are metabolic components that accompany congenital and immobility-related myopathies. The effects of immobility on metabolism have been previously observed. Physical inactivity has repeatedly been associated with insulin resistance and is caused by decreases in intramuscular glucose transporter 4 (GLUT-4) concentration [2]. As such, it may be unsurprising to find that altered metabolism may play a role in the deleterious effects of immobility on skeletal muscle. On the other hand, congenital myopathy is caused by defects in the structural proteins of muscle. There is little evidence which links metabolic processes to congenital myopathies, although certain therapies such as N-acetylcysteine have been studied to reduce the deleterious effects of oxidative stress on muscle damage in human recessive *RYR1*-related myopathies [27].

Relative to the other disease categories, inflammatory myositis and chronic systemic muscle diseases had much fewer enrichment terms in the GSEA analysis. As a result, we cannot confidently make any conclusions about the biological mechanisms of these diseases in comparison to other categories.

One limitation of our study design is that there may be confounding factors due to the heterogenous nature of multi-cohort studies. Since this study leverages data from many different studies, there is likely significant biological and non-biological heterogeneity that is ignored by our analysis. Although we chose to label the samples as belonging to one of six classes, the samples could be groupable by other unobserved disease subtypes. There may also be additional disease subtypes which are nested under our existing classes. This biological heterogeneity warrants further study as our analysis does not fully elucidate all the muscle disease subtypes which are contained in our dataset. Non-biological differences in the data may result in spurious differences in gene expression between muscle disease subtypes. When batch effects are not fully corrected, observed differences may be attributed to the characteristics of specific studies rather than the muscle disease subtypes.

Additionally, only the genes common among all studies were considered because using all the genes in our analysis would require non-trivial amounts of imputation. This results in the significant loss of information from genes that were dropped from the analysis. These dropped genes may contain important predictive information which cannot be recovered because of the challenges in comparing different microarray platforms.

The GSEA analysis conducted in this study should be interpreted with caution. The results from the analysis do not imply causative relationships between a biological pathway and a particular muscle disease. Such conclusions demand rigorous experimentation, and our results are primarily intended for mechanism discovery and hypothesis generation.

## Conclusion

In summary, we built a machine learning-based high-performing muscle disease classifier using multi-cohort microarray expression profile data. We also reported a gene signature containing 500 genes which is enriched in biological processes relevant to muscle weakness. There is overlap between our gene signature and gene signatures reported in the muscle disease literature. These findings are important because they address a gap in the difficulties of clinical muscle disease diagnosis. Muscle disease is highly heterogenous and difficult to classify. We demonstrate that utilizing expression profile data, machine learning algorithms, and data augmentation techniques can be an effective strategy for muscle disease classification. Our classification tool makes a novel addition to the muscle disease diagnostic toolbox and may fuel new interest in the analysis of gene expression profile data.

## Supplementary information

**Additional file 1.** Boxplots showing the gene expression distributions for samples before and after batch correction.

**Additional file 2.** Summary of gene expression datasets used in the study.

**Additional file 3.** Table of 500 genes (gene signature) used to train multiclass support vector machine muscle disease classifier.

**Additional file 4.** Table of top 5 upregulated biological processes for the 5 categories of muscle diseases

**Additional file 5.** Table of top 5 downregulated biological processes for the 5 categories of muscle diseases

**Additional file 6.** Enrichment map of the biological processes related to the top 500 genes identified by ANOVA included in the non-specific multiclass disease classifier

## Data Availability

We analyzed publicly available data sets. All of the data sets are listed in Additional file 2.

## Abbreviations

ICU: Intensive care unit
ICUAW: Acquired weakness
GEO: Gene expression omnibus
SMOTE: Synthetic minority oversampling technique
SVM: Support vector machine
AUC: Area under the receiver-operator curve
GSEA: Gene set enrichment analysis
CI: Confidence interval
GO: Gene ontology
BP: Biological Process
CIP: Critical illness polyneuropathy
CIM: Critical illness myopathy
CINM: Critical illness neuromyopathy
