# Supplementary material for: A machine learning-based clinical tool for diagnosing myopathy using multi-cohort microarray expression profiles
Source: J Transl Med. 2020 Nov 30;18:454. doi: 10.1186/s12967-020-02630-3 (PMC7708151; doi:10.1186/s12967-020-02630-3)
Supplement: Supplementary file 1 — Additional file 1. Boxplots showing the gene expression distributions for samples before and after batch correction. [file 12967_2020_2630_MOESM1_ESM.pdf]

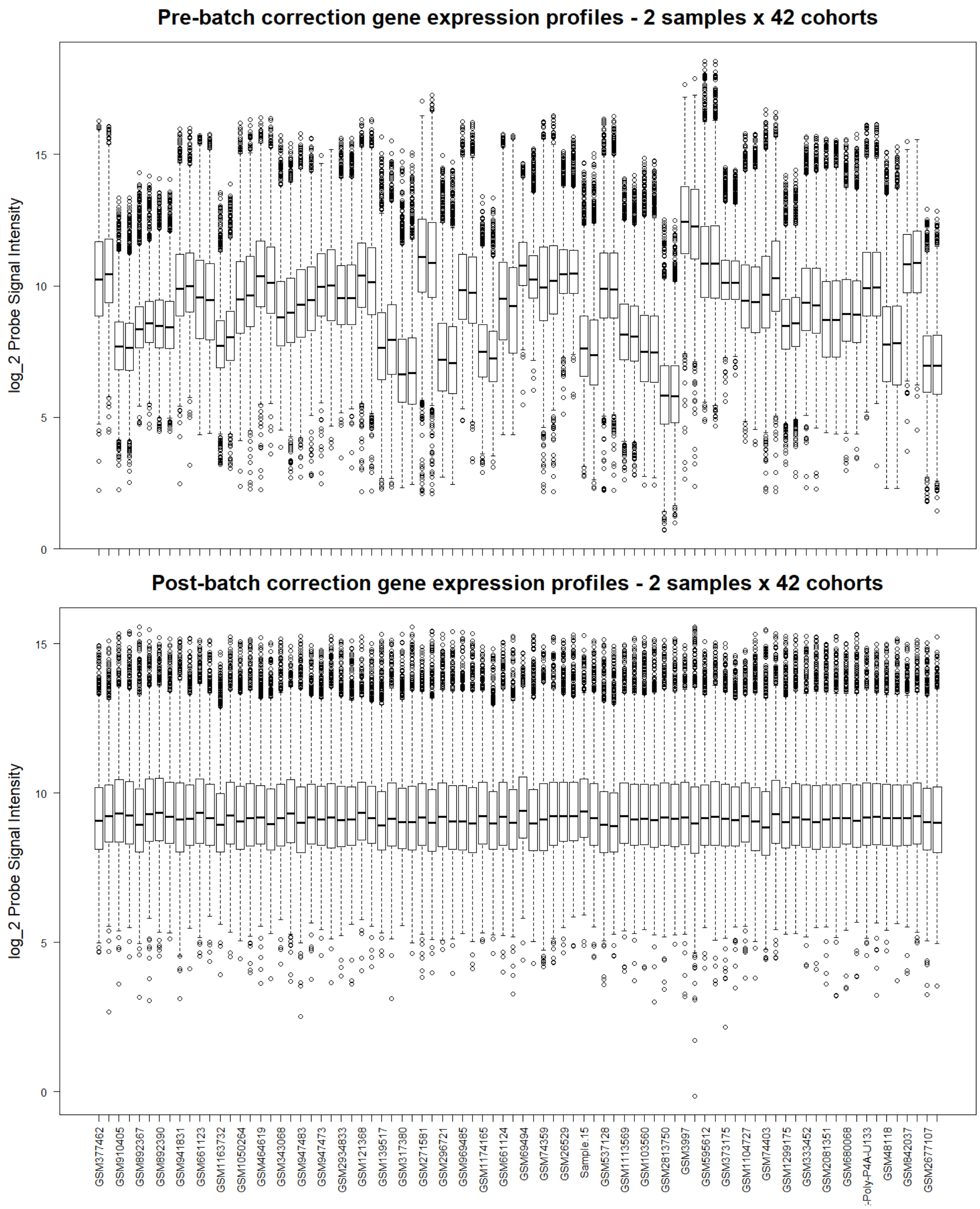

Supplemental Digital Content 1: Boxplots showing the gene expression distributions for samples before and after batch correction. Two samples were randomly sampled from each cohort (N = 84). It appears that the study-specific batch effects have been removed after batch correction since the means and variances are approximately the same across the samples. The rationale for batch correction is to account for batch effects. Batch effects are non-biological differences (e.g. the time of day that an assay is performed) that make samples from different batches difficult to compare. We corrected for batch effects using an empirical Bayes method (R package sva), which was demonstrated to be superior to other methods. Each patient cohort was considered a separate batch for batch correction.
