# Supplementary material for: A machine learning-based clinical tool for diagnosing myopathy using multi-cohort microarray expression profiles
Source: J Transl Med. 2020 Nov 30;18:454. doi: 10.1186/s12967-020-02630-3 (PMC7708151; doi:10.1186/s12967-020-02630-3)
Supplement: Supplementary file 2 — Additional file 2. Summary of gene expression datasets used in the study. [file 12967_2020_2630_MOESM2_ESM.pdf]

Supplemental Digital Content 2: Summary of gene expression datasets used in the study.

Reference	Cohort	Disease	Controls	Cases	Total	Platform
Arashiro	GSE15090	Congenital	5	5	10	GPL570
Perfetti	GSE37084	Congenital	10	10	20	GPL5175
Rahimov	GSE36398a	Congenital	16	8	24	GPL6244
Rahimov	GSE36398b	Congenital	8	10	18	GPL6244
Dorsey	GSE38417	Congenital	6	16	22	GPL570
Tasca	GSE26852	Congenital	7	12	19	GPL6947
Nakamori	GSE47968	Congenital	8	23	31	GPL5188
Screen	GSE42806	Congenital	5	7	12	GPL570
Voets	GSE18715	Congenital	12	6	18	GPL570
Bachinksi	GSE13608	Congenital	9	59	68	GPL570
Palermo	GSE38680a	Congenital	7	32	39	GPL570
Palermo	GSE38680b	Congenital	10	9	19	GPL570
Dadgar	GSE109178	Congenital	6	42	48	GPL570
Bakay	GSE3307	Congenital	13	66	79	GPL570
Pescatori	GSE6011	Congenital	14	23	37	GPL96
Eisenberg	GSE12648	Congenital	10	10	20	GPL96
Osborne	GSE10760	Congenital	60	38	98	GPL96
Saenz	GSE11681	Congenital	10	10	20	GPL96
Zhu	GSE39454	Infmyo	5	31	36	GPL570
Surez-Calvet	GSE48280	Infmyo	5	14	19	GPL6244
Tasca	GSE26852	Infmyo	7	7	14	GPL6947
Greenberg	GSE3112	Infmyo	11	29	40	GPL96
Bakay	GSE3307	Infmyo	13	21	34	GPL96
Greenberg	GSE1551	Infmyo	10	13	23	GPL96
Bernasconi	EMEXP2681	Infmyo	7	8	15	GPL96
Reich	GSE21496	Immobile	7	7	14	GPL570
Barres	GSE45745	Immobile	6	5	11	GPL13667
Urso	GSE5110	Immobile	5	5	10	GPL570
Rullman	GSE104999	Immobile	12	12	24	GPL17692
Park	GSE474	Immobile	8	16	24	GPL96
Alibegovic	GSE24215	Immobile	12	12	24	GPL6480
Abadi	GSE14901	Immobile	24	48	72	GPL570
Chen	GSE45462	Immobile	16	16	32	GPL570
Bakay	GSE3307	ICUAW	13	5	18	GPL96
Langhans	GSE53702	ICUAW	6	7	13	GPL5188
Fredriksson	GSE13205	ICUAW	8	13	21	GPL570
Walsh	GSE78929	ICUAW	8	24	32	GPL10558
Turan	GSE27536	Chronic	24	30	54	GPL570
Kreiner	EMTAB3671a	Chronic	12	12	24	GPL570
Radom-Aizik	GSE1786	Chronic	12	12	24	GPL96
Gallagher	GSE34111	Chronic	6	12	18	GPL570
Willis-Owen	GSE100281	Chronic	16	79	95	GPL11532
**TOTAL**			469	824	1293	

Abbreviations: Infmyo – inflammatory myositis, ICUAW – intensive care unit acquired weakness.
