# Supplementary material for: A machine learning-based clinical tool for diagnosing myopathy using multi-cohort microarray expression profiles
Source: J Transl Med. 2020 Nov 30;18:454. doi: 10.1186/s12967-020-02630-3 (PMC7708151; doi:10.1186/s12967-020-02630-3)
Supplement: Supplementary file 3 — Additional file 3. Table of 500 genes (gene signature) used to train multiclass support vector machine muscle disease classifier. [file 12967_2020_2630_MOESM3_ESM.pdf]

Supplemental Digital Content 3: List of 500 genes (gene signature) used to train multiclass support vector machine muscle disease classifier. F-statistic, p-values, and adjusted p-values using the Benjamini-Hochberg correction are reported. A Fisher's exact test was conducted to determine whether the gene signature was enriched with muscle specific genes. This test was conducted by creating 2x2 contingency tables with columns corresponding to gene signature membership and rows corresponding to muscle gene database membership. P-values were calculated under the null hypothesis of independence using a hypergeometric distribution. The muscle gene database used for determining muscle specificity was The Human Protein Atlas skeletal muscle-specific proteome (https://www.proteinatlas.org/humanproteome/tissue/skeletal%20muscle).

Gene	F-statistic	P-value	Adjusted P-value	Muscle Specific Gene? (Y/N)
**C1S**	26.281	0.000e+00	0.000e+00	N
**CD44**	14.8	1.115e-13	0.000e+00	N
**IQGAP1**	13.31	2.694e-12	0.000e+00	N
**CLTC**	13.217	3.286e-12	0.000e+00	N
**PTP4A1**	12.687	1.028e-11	0.000e+00	Y
**BZW1**	11.257	2.244e-10	0.000e+00	N
**ANXA5**	10.235	2.052e-09	0.000e+00	N
**CYB5R3**	9.322	1.488e-08	0.000e+00	N
**S100A10**	14.801	1.114e-13	6.177e-14	N
**PSAP**	12.677	1.049e-11	6.177e-14	N
**SDCBP**	11.498	1.333e-10	1.123e-13	N
**SERPING1**	14.896	9.082e-14	2.574e-13	N
**ANXA1**	13.869	8.136e-13	4.752e-13	N
**HTRA1**	10.643	8.477e-10	8.383e-13	N
**ENO1**	8.195	1.720e-07	1.647e-12	N
**ANXA4**	11.161	2.758e-10	2.973e-12	N
**COL6A3**	14.419	2.511e-13	3.634e-12	N
**ATP6AP2**	17.007	1.110e-15	4.753e-12	N
**LAMP1**	16.633	2.220e-15	6.145e-12	N
**ANXA2**	22.813	0.000e+00	1.263e-11	N
**TIMP1**	15.682	1.710e-14	1.410e-11	N
**HEXB**	15.405	3.075e-14	1.410e-11	N
**CELF2**	27	0.000e+00	1.519e-11	N
**CDKN1A**	17.602	2.220e-16	1.519e-11	N
**RGS2**	14.666	1.485e-13	1.653e-11	N
**S100A13**	12.869	6.946e-12	1.711e-11	N
**CCNG2**	10.868	5.208e-10	2.588e-11	N
**FAM13A**	11.536	1.229e-10	3.250e-11	N
**SAT1**	18.674	0.000e+00	3.291e-11	N
**GADD45A**	19.58	0.000e+00	4.460e-11	Y
**ACTC1**	22.359	0.000e+00	4.654e-11	N
**MN1**	13.993	6.246e-13	4.654e-11	Y
**CILP**	17.473	4.441e-16	4.693e-11	N
**CHRNA1**	19.218	0.000e+00	4.712e-11	Y
**IFI16**	12.304	2.340e-11	4.712e-11	N
**TGFBR2**	10.471	1.230e-09	4.827e-11	N
**CLIC1**	12.205	2.896e-11	5.024e-11	N
**CYFIP1**	11.979	4.722e-11	5.803e-11	N
**CAPN2**	8.436	1.019e-07	5.803e-11	N
**LGALS3**	10.878	5.093e-10	6.570e-11	N
**TUBA1A**	11.2	2.535e-10	7.037e-11	N
**TUBB6**	13.875	8.037e-13	8.413e-11	N
**CETN2**	14.046	5.567e-13	1.267e-10	N

**TUBA1C**	10.754	6.655e-10	1.267e-10	N
**PRKCQ**	12.782	8.379e-12	1.602e-10	Y
**C1R**	17.843	2.220e-16	1.629e-10	N
**OSBPL8**	15.26	4.197e-14	1.785e-10	N
**CAMK2G**	14.07	5.298e-13	1.905e-10	Y
**FTL**	10.304	1.765e-09	3.514e-10	N
**CFH**	12.238	2.698e-11	3.865e-10	N
**S100A6**	11.687	8.866e-11	4.571e-10	N
**C3**	19.093	0.000e+00	5.497e-10	N
**TMEM43**	11.919	5.374e-11	5.506e-10	N
**NDUFB11**	14.273	3.431e-13	5.737e-10	Y
**LRRC20**	9.759	5.763e-09	6.755e-10	Y
**DCUN1D2**	11.142	2.878e-10	7.343e-10	Y
**COL5A2**	10.471	1.229e-09	1.010e-09	N
**CTNNA1**	9.82	5.047e-09	1.122e-09	N
**CNDP2**	11.479	1.390e-10	1.259e-09	N
**CANX**	10.534	1.073e-09	1.259e-09	N
**CLIC4**	9.674	6.924e-09	1.259e-09	N
**SATB1**	7.818	3.901e-07	1.259e-09	N
**UCKL1**	14.115	4.809e-13	1.279e-09	N
**CAP1**	11.781	7.240e-11	1.460e-09	N
**ACTR3**	9.246	1.753e-08	1.537e-09	N
**TGFBI**	10.764	6.520e-10	1.567e-09	N
**IL1R1**	9.543	9.210e-09	1.961e-09	N
**VCAN**	12.137	3.359e-11	2.199e-09	N
**SCPEP1**	10.057	3.016e-09	2.919e-09	N
**CD58**	8.427	1.039e-07	3.177e-09	N
**CBFB**	11.46	1.447e-10	3.422e-09	N
**ATP1B3**	10.149	2.474e-09	3.426e-09	N
**LYZ**	10.666	8.064e-10	3.463e-09	N
**TMSB10**	12.362	2.068e-11	3.895e-09	N
**B2M**	11.694	8.732e-11	4.558e-09	N
**DUSP26**	8.738	5.295e-08	4.748e-09	Y
**DYNC1I2**	7.947	2.951e-07	4.956e-09	N
**ARPC5**	7.968	2.818e-07	5.095e-09	N
**PNMA1**	6.999	2.311e-06	5.222e-09	N
**LUM**	12.106	3.591e-11	5.625e-09	N
**CFL1**	9.314	1.514e-08	7.272e-09	N
**IFITM2**	8.131	1.980e-07	7.521e-09	N
**EFEMP1**	10.739	6.882e-10	8.395e-09	N
**COX6A2**	11.278	2.143e-10	8.446e-09	Y
**PHF11**	11.675	9.087e-11	8.578e-09	N
**NES**	9.437	1.158e-08	8.578e-09	N
**PON2**	7.447	8.743e-07	8.578e-09	N
**SH3BGRL**	8.59	7.289e-08	8.621e-09	N
**SH3BP5**	11.17	2.710e-10	8.800e-09	N
**SGCA**	7.799	4.069e-07	8.800e-09	Y
**EIF3D**	12.923	6.189e-12	9.044e-09	N
**RAB31**	10.475	1.218e-09	1.018e-08	N
**BZW2**	9.545	9.174e-09	1.093e-08	Y
**TUBA1B**	9.893	4.307e-09	1.143e-08	N
**VIM**	9.215	1.878e-08	1.160e-08	N
**HLA.DPB1**	7.062	2.012e-06	1.402e-08	N
**YWHAZ**	10.209	2.172e-09	1.446e-08	N
**LIMA1**	10.479	1.210e-09	1.464e-08	N
**IDH2**	16.335	4.219e-15	1.558e-08	Y
**GNS**	10.349	1.600e-09	1.588e-08	N
**DYNLL1**	8.666	6.179e-08	1.778e-08	N
**ZFP36L1**	13.961	6.681e-13	1.798e-08	N

**MGP**	10.68	7.822e-10	1.841e-08	N
**ANGPTL2**	13.457	1.964e-12	2.027e-08	N
**PREPL**	11.443	1.502e-10	2.039e-08	N
**ARHGAP1**	8.321	1.309e-07	2.039e-08	N
**MACF1**	11.51	1.297e-10	2.077e-08	N
**GBP2**	12.227	2.766e-11	2.127e-08	N
**MAPRE1**	7.837	3.747e-07	2.144e-08	N
**ZFP36L2**	7.205	1.478e-06	2.192e-08	N
**RND3**	10.679	7.843e-10	2.307e-08	N
**HIGD2A**	9.159	2.118e-08	2.412e-08	N
**PITPNB**	8.415	1.068e-07	2.618e-08	N
**LAMA2**	13.448	2.004e-12	2.843e-08	N
**PRKAR1A**	8.822	4.410e-08	2.843e-08	N
**TUBB2A**	8.477	9.326e-08	2.843e-08	N
**MSN**	8.04	2.411e-07	2.843e-08	N
**GSN**	7.673	5.346e-07	2.843e-08	N
**PDCD6IP**	6.361	9.176e-06	2.843e-08	N
**PRNP**	8.64	6.539e-08	2.920e-08	N
**CTSB**	7.62	6.000e-07	3.287e-08	N
**MFN2**	14.027	5.806e-13	3.337e-08	Y
**CAPRIN2**	14.295	3.271e-13	3.533e-08	N
**ARHGDIB**	7.451	8.658e-07	3.604e-08	N
**COL6A1**	9.553	9.015e-09	3.836e-08	N
**RPL3L**	14.727	1.303e-13	4.120e-08	Y
**RPS27L**	14.017	5.929e-13	4.183e-08	N
**FBLN5**	7.941	2.990e-07	4.358e-08	N
**PTEN**	10.479	1.208e-09	4.533e-08	N
**EPM2A**	9.879	4.434e-09	4.533e-08	Y
**LASP1**	8.642	6.522e-08	4.533e-08	N
**ATP1A1**	8.811	4.510e-08	5.098e-08	N
**COL1A1**	9.898	4.263e-09	5.341e-08	N
**THBS4**	8.027	2.481e-07	5.689e-08	Y
**TRAM1**	10.268	1.910e-09	5.875e-08	N
**HIF1A**	10.037	3.149e-09	5.941e-08	N
**PPT1**	8.023	2.500e-07	5.941e-08	N
**RAD23A**	8.897	3.744e-08	5.952e-08	Y
**IFITM1**	8.811	4.512e-08	7.166e-08	N
**APLP2**	8.18	1.778e-07	8.235e-08	N
**CST3**	6.917	2.758e-06	8.264e-08	N
**GOT2**	14.724	1.310e-13	8.449e-08	Y
**CREG1**	7.172	1.587e-06	8.948e-08	N
**HLA.DPA1**	8.373	1.169e-07	9.041e-08	N
**ATP5D**	9.819	5.061e-09	9.387e-08	N
**TRIP10**	9.553	9.015e-09	9.387e-08	Y
**CD81**	8.778	4.850e-08	9.839e-08	N
**MAPK1**	11.124	2.991e-10	1.033e-07	N
**ACLY**	7.238	1.376e-06	1.041e-07	N
**LRP10**	9.481	1.052e-08	1.133e-07	N
**RNF123**	11.027	3.692e-10	1.170e-07	Y
**ARHGEF6**	10.467	1.240e-09	1.170e-07	N
**DSTN**	7.229	1.403e-06	1.219e-07	N
**SEPT9**	8.032	2.451e-07	1.333e-07	N
**HRASLS**	8.765	4.989e-08	1.522e-07	Y
**UBE2D1**	8.838	4.257e-08	1.548e-07	Y
**SRGN**	8.47	9.468e-08	1.548e-07	N
**PGAM2**	12.09	3.717e-11	1.553e-07	Y
**GPD1L**	9.791	5.376e-09	1.553e-07	Y
**NQO1**	7.148	1.673e-06	1.553e-07	N
**IRAK1**	8.263	1.484e-07	1.592e-07	N

**HPRT1**	8.891	3.795e-08	1.725e-07	N
**SRP72**	10.257	1.955e-09	1.742e-07	N
**ARL6IP5**	8.449	9.915e-08	1.817e-07	N
**MACROD1**	9.06	2.627e-08	1.885e-07	Y
**GMFB**	6.771	3.786e-06	1.885e-07	N
**GNG12**	8.907	3.663e-08	2.407e-07	N
**TM9SF2**	6.352	9.372e-06	2.434e-07	N
**DPT**	10.206	2.183e-09	2.446e-07	N
**PPP4R1**	11.169	2.713e-10	2.476e-07	N
**MTSS1**	13.328	2.592e-12	2.478e-07	N
**PFKFB1**	8.554	7.886e-08	2.531e-07	Y
**PYGM**	10.989	4.002e-10	2.718e-07	Y
**SMARCA4**	10.209	2.171e-09	2.725e-07	N
**ARMCX1**	11.064	3.404e-10	2.902e-07	N
**TFPI**	9.965	3.683e-09	3.256e-07	N
**TM9SF3**	9.286	1.610e-08	4.138e-07	N
**LMCD1**	9.566	8.753e-09	4.248e-07	Y
**PTPN3**	14.065	5.353e-13	4.597e-07	Y
**MMP2**	10.576	9.797e-10	5.046e-07	N
**SHC1**	7.878	3.430e-07	5.052e-07	N
**SAE1**	7.385	9.999e-07	5.052e-07	N
**KANK2**	6.179	1.362e-05	5.052e-07	N
**MDH2**	10.834	5.602e-10	5.363e-07	Y
**LITAF**	10.448	1.291e-09	5.453e-07	N
**ADAM10**	6.764	3.844e-06	5.498e-07	N
**PSME1**	7.096	1.869e-06	5.963e-07	N
**AKR1C3**	6.224	1.234e-05	6.104e-07	N
**STAT6**	7.306	1.186e-06	6.291e-07	N
**NREP**	11.144	2.862e-10	6.308e-07	N
**PNPLA4**	10.601	9.286e-10	6.308e-07	N
**GRB10**	9.765	5.683e-09	6.308e-07	N
**DCN**	9.697	6.586e-09	6.308e-07	N
**PXMP2**	9.301	1.557e-08	6.436e-07	N
**PABPC3**	7.969	2.812e-07	6.436e-07	N
**GAMT**	10.897	4.888e-10	6.504e-07	Y
**KLHDC3**	15.99	8.882e-15	6.717e-07	Y
**TNFRSF1A**	7.736	4.667e-07	6.717e-07	N
**PRCP**	6.876	3.015e-06	6.737e-07	N
**TMEM87A**	11.735	7.993e-11	6.946e-07	N
**FYCO1**	8.797	4.650e-08	6.946e-07	Y
**CD9**	6.673	4.674e-06	6.949e-07	N
**CD47**	8.945	3.374e-08	6.982e-07	N
**COX7A1**	6.231	1.216e-05	6.984e-07	Y
**STK38**	6.438	7.781e-06	7.236e-07	N
**CACNA1S**	6.146	1.461e-05	7.381e-07	Y
**CYC1**	13.799	9.446e-13	7.419e-07	Y
**ENO3**	8.356	1.212e-07	7.645e-07	Y
**DHPS**	8.2	1.704e-07	7.922e-07	N
**CTTN**	8.24	1.562e-07	8.214e-07	N
**MCTS1**	7.36	1.055e-06	8.214e-07	N
**FHL3**	7.227	1.408e-06	8.214e-07	Y
**SLC25A12**	9.298	1.568e-08	8.973e-07	Y
**PGRMC1**	6.947	2.581e-06	9.716e-07	N
**SDC2**	7.261	1.309e-06	9.821e-07	N
**PDK2**	10.073	2.914e-09	1.123e-06	Y
**RHOA**	6.377	8.872e-06	1.135e-06	N
**FBLN1**	6.348	9.449e-06	1.184e-06	N
**FBN1**	10.091	2.802e-09	1.211e-06	N
**RGS3**	15.561	2.220e-14	1.230e-06	N

**STAM**	10.124	2.611e-09	1.259e-06	N
**STAT1**	8.851	4.137e-08	1.277e-06	N
**DIP2C**	6.31	1.026e-05	1.306e-06	N
**CHMP5**	10.379	1.499e-09	1.312e-06	N
**COX6B1**	6.182	1.353e-05	1.332e-06	N
**VAMP8**	6.42	8.081e-06	1.339e-06	N
**UBE2D4**	6.38	8.815e-06	1.343e-06	Y
**CS**	12.22	2.805e-11	1.347e-06	Y
**DSE**	8.813	4.492e-08	1.347e-06	N
**SP100**	8.601	7.118e-08	1.365e-06	N
**ARF4**	6.618	5.272e-06	1.395e-06	N
**SPAG7**	12.518	1.478e-11	1.573e-06	Y
**HNRNPR**	9.459	1.104e-08	1.586e-06	N
**CALD1**	6.502	6.774e-06	1.592e-06	N
**GYPC**	6.961	2.507e-06	1.602e-06	N
**TRIM38**	7.078	1.946e-06	1.619e-06	N
**ATP2B2**	6.129	1.516e-05	1.643e-06	Y
**TMCO1**	6.568	5.868e-06	1.691e-06	N
**LMNA**	7.15	1.664e-06	1.840e-06	N
**ELOVL5**	6.328	9.857e-06	1.895e-06	N
**GUSB**	6.685	4.561e-06	2.102e-06	N
**RAB11A**	9.489	1.036e-08	2.229e-06	N
**CKAP4**	7.315	1.163e-06	2.410e-06	N
**RAB13**	6.271	1.117e-05	2.531e-06	N
**ATP5H**	8.814	4.484e-08	2.588e-06	N
**GNAI3**	8.365	1.191e-07	2.588e-06	N
**ACADM**	8.735	5.322e-08	2.731e-06	Y
**ASPN**	7.668	5.406e-07	2.872e-06	N
**CNN3**	7.065	2.002e-06	2.872e-06	N
**ETF1**	8.274	1.448e-07	2.924e-06	N
**ADSL**	6.715	4.272e-06	2.986e-06	Y
**MARCKS**	6.665	4.763e-06	2.999e-06	N
**GNAQ**	7.443	8.814e-07	3.117e-06	N
**EMP3**	6.494	6.887e-06	3.168e-06	N
**COX10**	11.192	2.580e-10	3.383e-06	Y
**CEP85**	11.173	2.691e-10	3.383e-06	Y
**EMP1**	8.37	1.178e-07	3.671e-06	N
**HNRNPAB**	6.998	2.313e-06	3.872e-06	N
**FBXO3**	8.742	5.245e-08	3.944e-06	N
**AGFG1**	11.615	1.036e-10	3.955e-06	N
**AKAP12**	6.77	3.792e-06	4.106e-06	N
**PICALM**	7.995	2.660e-07	4.461e-06	N
**TMEM59**	7.698	5.063e-07	4.498e-06	N
**PHYH**	8.543	8.084e-08	4.655e-06	Y
**CYR61**	8.278	1.437e-07	4.765e-06	N
**SEPT11**	6.307	1.031e-05	4.774e-06	N
**ACO2**	12.565	1.336e-11	4.991e-06	Y
**PHB2**	7.796	4.095e-07	4.991e-06	N
**MAP4K4**	6.131	1.508e-05	5.030e-06	N
**TIMP3**	6.426	7.975e-06	5.149e-06	N
**SESN1**	6.771	3.785e-06	5.159e-06	N
**RASGRP3**	6.603	5.446e-06	5.261e-06	Y
**PDLIM2**	8.798	4.642e-08	5.491e-06	N
**CCNG1**	6.241	1.189e-05	5.506e-06	N
**ZNF672**	9.543	9.198e-09	5.540e-06	N
**DDAH1**	7.198	1.501e-06	5.540e-06	N
**SLC29A1**	8.771	4.926e-08	6.064e-06	N
**MYOZ3**	8.79	4.723e-08	6.219e-06	Y
**HLTF**	7.22	1.430e-06	6.219e-06	N

**KCTD12**	6.722	4.205e-06	6.473e-06	N
**TLE1**	7.654	5.571e-07	6.744e-06	N
**YWHAB**	7.652	5.600e-07	7.639e-06	N
**ADD3**	6.475	7.184e-06	7.639e-06	N
**MRS2**	6.409	8.287e-06	8.639e-06	N
**NONO**	8.404	1.092e-07	8.643e-06	N
**ALDOA**	8.023	2.502e-07	8.807e-06	Y
**SIRT2**	14.631	1.599e-13	8.896e-06	Y
**DYNLT3**	6.717	4.257e-06	9.443e-06	N
**SUCLG1**	10.061	2.990e-09	9.808e-06	N
**MLLT11**	10.465	1.247e-09	9.941e-06	N
**MRPL48**	6.365	9.099e-06	9.941e-06	N
**GLRX**	8.55	7.963e-08	1.109e-05	Y
**SLC25A3**	10.628	8.747e-10	1.166e-05	N
**ADAR**	6.2	1.301e-05	1.166e-05	N
**SAMM50**	6.35	9.409e-06	1.199e-05	N
**NDUFS3**	13.257	3.016e-12	1.210e-05	Y
**MYH7**	7.532	7.259e-07	1.288e-05	Y
**ANP32B**	9.309	1.530e-08	1.292e-05	N
**TPI1**	9.029	2.810e-08	1.311e-05	Y
**SLC39A6**	8.197	1.714e-07	1.311e-05	N
**TCF7L2**	7.165	1.612e-06	1.311e-05	N
**ABCA1**	6.874	3.025e-06	1.311e-05	N
**AKAP1**	8.27	1.462e-07	1.315e-05	Y
**MRPS30**	9.696	6.603e-09	1.332e-05	N
**IL13RA1**	7.548	7.017e-07	1.349e-05	N
**ACAT1**	9.717	6.314e-09	1.450e-05	N
**CHCHD3**	7.692	5.138e-07	1.452e-05	Y
**KCNS3**	7.731	4.721e-07	1.508e-05	Y
**UBE2L6**	6.478	7.131e-06	1.519e-05	N
**PGM1**	7.133	1.726e-06	1.543e-05	Y
**EML1**	7.713	4.902e-07	1.549e-05	N
**AP3S1**	7.373	1.026e-06	1.647e-05	N
**GDI2**	9.85	4.728e-09	1.749e-05	N
**CXCL14**	8.747	5.189e-08	1.749e-05	N
**NSMCE4A**	9.842	4.810e-09	1.773e-05	N
**TPPP3**	6.488	6.983e-06	1.854e-05	N
**TMX1**	8.382	1.147e-07	1.915e-05	N
**SLC16A1**	8.366	1.187e-07	2.054e-05	N
**PLP2**	10.471	1.230e-09	2.081e-05	N
**SCN1B**	6.278	1.099e-05	2.102e-05	Y
**WDR1**	7.611	6.114e-07	2.193e-05	N
**FN1**	7.878	3.424e-07	2.277e-05	N
**HSPB11**	8.377	1.159e-07	2.517e-05	N
**RIN2**	6.99	2.352e-06	2.532e-05	N
**UBE2B**	6.91	2.799e-06	2.552e-05	Y
**WASF2**	6.649	4.930e-06	2.665e-05	N
**NAP1L1**	10.69	7.651e-10	2.678e-05	N
**VAT1**	6.467	7.310e-06	2.678e-05	N
**MYOM1**	7.555	6.914e-07	2.691e-05	Y
**CISD1**	7.554	6.930e-07	2.700e-05	N
**SLC25A4**	12.224	2.780e-11	2.731e-05	Y
**CCT2**	7.383	1.003e-06	2.939e-05	N
**TRMT112**	9.529	9.497e-09	2.960e-05	N
**MAPK12**	6.527	6.409e-06	2.960e-05	Y
**LRRC2**	6.798	3.568e-06	3.211e-05	Y
**FBP2**	6.231	1.217e-05	3.324e-05	Y
**DDX50**	6.279	1.096e-05	3.354e-05	N
**TCF12**	6.982	2.394e-06	3.364e-05	N

**COX5A**	8.769	4.950e-08	3.384e-05	Y
**MAGED2**	8.685	5.935e-08	3.560e-05	N
**RAP1B**	7.334	1.117e-06	3.590e-05	N
**BHLHE41**	6.196	1.313e-05	3.592e-05	Y
**OXA1L**	7.446	8.759e-07	3.887e-05	N
**THAP11**	8.654	6.350e-08	3.913e-05	N
**EIF4E2**	8.653	6.367e-08	3.987e-05	N
**HADH**	8.064	2.287e-07	4.036e-05	N
**COX5B**	8.937	3.432e-08	4.195e-05	Y
**TGOLN2**	7.757	4.454e-07	4.244e-05	N
**PMP22**	9.807	5.194e-09	4.487e-05	N
**TP63**	8.392	1.122e-07	4.616e-05	Y
**COX8A**	8.409	1.082e-07	4.686e-05	Y
**PPP1R3C**	6.611	5.345e-06	4.943e-05	Y
**DLG5**	6.897	2.879e-06	4.953e-05	N
**SNUPN**	7.851	3.634e-07	5.110e-05	N
**ACTN2**	8.325	1.296e-07	5.121e-05	Y
**DLD**	6.199	1.302e-05	5.147e-05	N
**ECHDC2**	10.337	1.645e-09	5.638e-05	N
**UQCRFS1**	9.25	1.739e-08	5.656e-05	Y
**RHOBTB1**	6.492	6.919e-06	5.660e-05	Y
**ATF4**	6.807	3.501e-06	5.740e-05	N
**MDH1**	9.438	1.157e-08	5.785e-05	N
**SYPL1**	7.152	1.656e-06	5.816e-05	N
**PIK3C2B**	6.445	7.656e-06	6.168e-05	N
**ECSIT**	7.92	3.126e-07	6.444e-05	N
**MLEC**	7.617	6.036e-07	6.501e-05	N
**MAP1B**	6.714	4.278e-06	6.505e-05	N
**DUSP13**	6.613	5.330e-06	6.567e-05	Y
**MYL3**	8.637	6.584e-08	6.923e-05	Y
**WDR45B**	6.573	5.813e-06	6.982e-05	N
**GTPBP8**	6.874	3.027e-06	7.026e-05	N
**ROCK1**	7.568	6.713e-07	7.064e-05	N
**ATP5O**	7.162	1.621e-06	7.064e-05	N
**FKBP3**	6.549	6.120e-06	7.078e-05	Y
**IDH3B**	6.642	5.000e-06	7.389e-05	N
**NDUFV1**	13.756	1.037e-12	7.407e-05	Y
**COQ3**	7.943	2.977e-07	7.407e-05	N
**SLC25A11**	10.063	2.978e-09	7.453e-05	Y
**CLIC5**	6.323	9.973e-06	7.650e-05	Y
**BLVRB**	6.646	4.958e-06	8.301e-05	N
**PPP1R1A**	8.705	5.678e-08	8.435e-05	Y
**LPAR1**	8.388	1.132e-07	8.872e-05	N
**FH**	8.019	2.523e-07	9.116e-05	N
**SIRT5**	10.821	5.764e-10	9.274e-05	N
**THYN1**	7.251	1.336e-06	9.276e-05	N
**MGEA5**	6.555	6.034e-06	9.368e-05	N
**MPC1**	8.638	6.580e-08	9.637e-05	N
**TAX1BP1**	6.929	2.688e-06	9.739e-05	N
**ATP6V1A**	7.14	1.699e-06	1.015e-04	N
**ATP5G1**	7.447	8.745e-07	1.092e-04	N
**BCL2**	6.418	8.118e-06	1.143e-04	N
**ATP2A2**	7.049	2.072e-06	1.157e-04	Y
**ATP5A1**	8.937	3.431e-08	1.201e-04	N
**ATP5B**	12.649	1.113e-11	1.220e-04	N
**AHNAK**	7.073	1.965e-06	1.230e-04	N
**ABI1**	6.236	1.202e-05	1.239e-04	N
**RAB22A**	6.188	1.334e-05	1.279e-04	N
**MRPL15**	7.641	5.730e-07	1.280e-04	Y

**NDUFB7**	8.255	1.512e-07	1.314e-04	Y
**MFAP5**	7.151	1.662e-06	1.375e-04	N
**WSB1**	6.98	2.404e-06	1.377e-04	N
**HCCS**	6.241	1.189e-05	1.410e-04	N
**RRAGC**	8.345	1.243e-07	1.446e-04	N
**MYOT**	6.903	2.843e-06	1.562e-04	Y
**DLAT**	8.102	2.107e-07	1.580e-04	N
**HAX1**	6.965	2.486e-06	1.600e-04	N
**NNT**	8.938	3.426e-08	1.637e-04	Y
**COX4I1**	8.746	5.199e-08	1.672e-04	N
**NDUFA10**	7.777	4.266e-07	1.677e-04	N
**COX7B**	6.227	1.227e-05	1.701e-04	N
**IRS1**	6.767	3.820e-06	1.815e-04	N
**TNNT1**	6.675	4.655e-06	1.815e-04	Y
**TNNC1**	6.387	8.688e-06	1.818e-04	Y
**PSMG2**	7.188	1.532e-06	2.139e-04	N
**AES**	6.171	1.383e-05	2.142e-04	N
**RAB5A**	8.524	8.420e-08	2.247e-04	N
**ETS2**	6.979	2.410e-06	2.269e-04	N
**SDHB**	8.99	3.057e-08	2.408e-04	N
**CLPP**	7.94	2.997e-07	2.494e-04	N
**ETFDH**	6.856	3.151e-06	2.686e-04	N
**MYCBP2**	7.958	2.879e-07	2.687e-04	N
**ERP44**	7.06	2.021e-06	2.780e-04	N
**UQCRC1**	13.661	1.270e-12	2.790e-04	Y
**PDGFRL**	6.344	9.524e-06	2.805e-04	N
**HGSNAT**	7.387	9.960e-07	2.951e-04	N
**NDUFC1**	7.613	6.097e-07	2.957e-04	Y
**CKM**	7.674	5.341e-07	2.965e-04	Y
**MT2A**	6.568	5.873e-06	3.490e-04	N
**CCDC28B**	7.257	1.319e-06	3.541e-04	Y
**TCAP**	6.44	7.747e-06	3.648e-04	Y
**IGF1**	6.363	9.149e-06	3.745e-04	N
**ARHGEF12**	7.199	1.497e-06	3.820e-04	N
**TACO1**	8.701	5.734e-08	3.848e-04	N
**POLR2G**	6.352	9.371e-06	3.848e-04	N
**PLCL1**	6.275	1.106e-05	3.902e-04	N
**CKMT2**	11.001	3.903e-10	4.630e-04	Y
**TRIP12**	6.248	1.172e-05	4.704e-04	N
**ALDH5A1**	6.788	3.644e-06	5.234e-04	N
**PTP4A3**	7.367	1.039e-06	5.285e-04	Y
**TLN2**	6.665	4.764e-06	5.606e-04	N
**PPIF**	11.403	1.638e-10	6.063e-04	N
**VDAC1**	7.029	2.161e-06	6.188e-04	Y
**MAP3K7**	7.809	3.980e-07	6.288e-04	N
**COQ9**	10.39	1.465e-09	6.698e-04	Y
**EHBP1**	6.49	6.953e-06	6.977e-04	N
**KALRN**	6.35	9.405e-06	7.448e-04	N
**COA7**	6.399	8.467e-06	7.920e-04	N
**PJA2**	6.548	6.137e-06	8.726e-04	N
**RPL31**	6.307	1.033e-05	9.563e-04	N
**GNL2**	7.701	5.036e-07	9.750e-04	N
**UQCR10**	6.558	6.000e-06	1.175e-03	Y
**PKM**	6.132	1.506e-05	1.379e-03	Y
**GOT1**	9.63	7.618e-09	1.440e-03	Y
**ANK1**	7.153	1.653e-06	1.442e-03	Y
**DCTPP1**	6.528	6.407e-06	1.507e-03	N
**ARID4B**	6.351	9.377e-06	1.883e-03	N
**NDUFA13**	6.284	1.084e-05	1.899e-03	N

**HERC1**	6.231	1.217e-05	2.055e-03	N
**GATM**	7.51	7.612e-07	2.127e-03	N
**MRPS7**	6.776	3.745e-06	2.137e-03	N
**GLRX5**	10.651	8.333e-10	2.219e-03	N
**SEL1L**	6.211	1.270e-05	2.311e-03	N
**NDUFA8**	7.911	3.188e-07	2.343e-03	N
**DCP2**	7.098	1.861e-06	2.361e-03	N
**ATP5G3**	8.717	5.540e-08	2.381e-03	N
**GYS1**	6.307	1.033e-05	2.390e-03	Y
**OFD1**	6.202	1.293e-05	2.490e-03	N
**MCM6**	6.317	1.010e-05	2.511e-03	N
**MRPL2**	6.654	4.874e-06	2.612e-03	N
**ETFB**	7.488	7.991e-07	2.636e-03	N
**RPP14**	6.566	5.893e-06	2.849e-03	Y
**TCF4**	7.307	1.183e-06	3.155e-03	N
**ZC3H7A**	7.023	2.194e-06	3.173e-03	N
**AUH**	6.415	8.178e-06	3.415e-03	N
**MAN1A2**	6.226	1.229e-05	3.566e-03	N
**MSRB2**	6.924	2.715e-06	3.612e-03	N
**MRPL16**	6.718	4.248e-06	3.687e-03	N
**NMRK2**	7.657	5.544e-07	4.311e-03	Y
**COA1**	6.373	8.942e-06	4.541e-03	N
**GRSF1**	6.139	1.483e-05	6.259e-03	Y
**UBE2E1**	9.044	2.722e-08	6.794e-03	N
**SMIM7**	8.592	7.265e-08	7.021e-03	N
**SDHA**	6.803	3.532e-06	7.030e-03	Y
**MT1G**	6.158	1.423e-05	1.040e-02	N
**CDK16**	8.284	1.420e-07	1.160e-02	Y
**NDUFS2**	8.744	5.218e-08	1.278e-02	Y
**PSMB1**	6.521	6.498e-06	1.433e-02	N
**PDHA1**	6.421	8.072e-06	1.490e-02	Y
**CRAT**	7.233	1.389e-06	1.566e-02	Y
**UBAC1**	6.287	1.079e-05	1.611e-02	Y
**DBI**	6.254	1.157e-05	1.611e-02	N
**HSPB6**	7.349	1.080e-06	1.933e-02	Y
**PRRC2C**	6.823	3.380e-06	1.954e-02	N
**ABCA5**	7.53	7.298e-07	2.212e-02	Y
**AIFM1**	7.219	1.433e-06	2.656e-02	N
**G0S2**	6.757	3.898e-06	3.010e-02	N
**GNG5**	7.232	1.393e-06	3.562e-02	N
**SMAD1**	6.262	1.138e-05	3.651e-02	N
**PSMA4**	6.341	9.595e-06	7.091e-02	N
**SFPQ**	6.273	1.111e-05	8.704e-02	N
**ACADVL**	7.336	1.111e-06	1.245e-01	N
