# Supplementary material for: A machine learning-based clinical tool for diagnosing myopathy using multi-cohort microarray expression profiles
Source: J Transl Med. 2020 Nov 30;18:454. doi: 10.1186/s12967-020-02630-3 (PMC7708151; doi:10.1186/s12967-020-02630-3)
Supplement: Supplementary file 4 — Additional file 4. Table of top 5 upregulated biological processes for the 5 categories of muscle diseases [file 12967_2020_2630_MOESM4_ESM.pdf]

Supplemental Content 4: Top 5 upregulated biological processes for the 5 categories of muscle diseases. Only significant terms (FDR q-val < 0.05) were included in the table. * Infmyo has no significant upregulated biological processes.

Disease*	Name	Size	ES	NES	FDR q-val
Chronic	Regulation of viral genome replication	7	0.76	2.115	0.035
Chronic	Kidney epithelium development	6	0.736	2.032	0.047
Chronic	Development of primary sexual characteristics	5	0.711	1.863	0.049
Congenital	Cellular respiration	44	0.547	2.839	<0.001
Congenital	Generation of precursor metabolites and energy	68	0.456	2.71	<0.001
Congenital	Dicarboxylic acid metabolic process	14	0.713	2.471	<0.001
Congenital	Energy derivation by oxidation of organic compounds	52	0.509	2.44	<0.001
Congenital	Aerobic respiration	27	0.574	2.263	<0.001
ICUAW	Neural precursor cell proliferation	7	0.869	2.133	<0.001
ICUAW	Cardiac muscle tissue morphogenesis	6	0.783	2.085	<0.001
ICUAW	Positive regulation of cell cycle process	10	0.756	2.044	0.016
ICUAW	Negative regulation of phosphorylation	24	0.506	1.945	0.019
ICUAW	Muscle organ development	22	0.615	1.999	0.024
Immobile	Energy derivation by oxidation of organic compounds	52	0.65	3.933	<0.001
Immobile	Oxidation reduction process	81	0.548	3.487	<0.001
Immobile	Generation of precursor metabolites and energy	68	0.593	2.851	<0.001
Immobile	ATP metabolic process	40	0.584	2.82	<0.001
Immobile	Electron transport chain	34	0.54	2.792	<0.001

Abbreviations: Infmyo – inflammatory myositis, ICUAW – intensive care unit acquired weakness.
