# Supplementary material for: A machine learning-based clinical tool for diagnosing myopathy using multi-cohort microarray expression profiles
Source: J Transl Med. 2020 Nov 30;18:454. doi: 10.1186/s12967-020-02630-3 (PMC7708151; doi:10.1186/s12967-020-02630-3)
Supplement: Supplementary file 5 — Additional file 5. Table of top 5 downregulated biological processes for the 5 categories of muscle diseases [file 12967_2020_2630_MOESM5_ESM.pdf]

Supplemental Content 5: Top 5 downregulated biological pathways for 5 categories of muscle disease. Only significant terms (FDR q-val < 0.05) were included in the table. * ICUAW has no significant downregulated biological processes.

**Disease***	**Name**	**Size**	**ES**	**NES**	**FDR q-val**
Chronic	Cytosolic transport	7	-0.812	-1.903	0.036
Chronic	Cellular component assembly involved in morphogenesis	10	-0.761	-1.879	0.043
Chronic	Regionalization	9	-0.696	-1.824	0.049
Congenital	Immune effector process	70	-0.56	-2.171	<0.001
Congenital	Cytokine mediated signaling pathway	40	-0.568	-2.166	<0.001
Congenital	Cytoskeleton organization	74	-0.503	-2.155	<0.001
Congenital	Regulation of apoptotic signaling pathway	25	-0.587	-2.127	<0.001
Congenital	Regulation of peptidyl tyrosine phosphorylation	11	-0.642	-1.934	0.005
Immobile	Posttranscriptional regulation of gene expression	26	-0.639	-2.45	<0.001
Immobile	Regulation of immune response	40	-0.569	-2.273	<0.001
Immobile	Defense response to other organism	42	-0.557	-2.231	<0.001
Immobile	Regulation of cell junction assembly	9	-0.776	-2.174	<0.001
Immobile	Regulation of immune system process	60	-0.552	-2.142	<0.001
Infmyo	Muscle cell proliferation	12	-0.595	-2.609	<0.001
Infmyo	Developmental cell growth	10	-0.584	-2.338	<0.001
Infmyo	Positive regulation of growth	13	-0.502	-2.255	<0.001
Infmyo	Smooth muscle cell proliferation	10	-0.623	-2.137	<0.001
Infmyo	Positive regulation of cell cycle	12	-0.567	-2.018	0.006

Abbreviations: Infmyo – inflammatory myositis, ICUAW – intensive care unit acquired weakness.
