# Supplementary material for: A machine learning-based clinical tool for diagnosing myopathy using multi-cohort microarray expression profiles
Source: J Transl Med. 2020 Nov 30;18:454. doi: 10.1186/s12967-020-02630-3 (PMC7708151; doi:10.1186/s12967-020-02630-3)
Supplement: Supplementary file 6 — Additional file 6. Enrichment map of the biological processes related to the top 500 genes identified by ANOVA included in the non-specific multiclass disease classifier [file 12967_2020_2630_MOESM6_ESM.pdf]

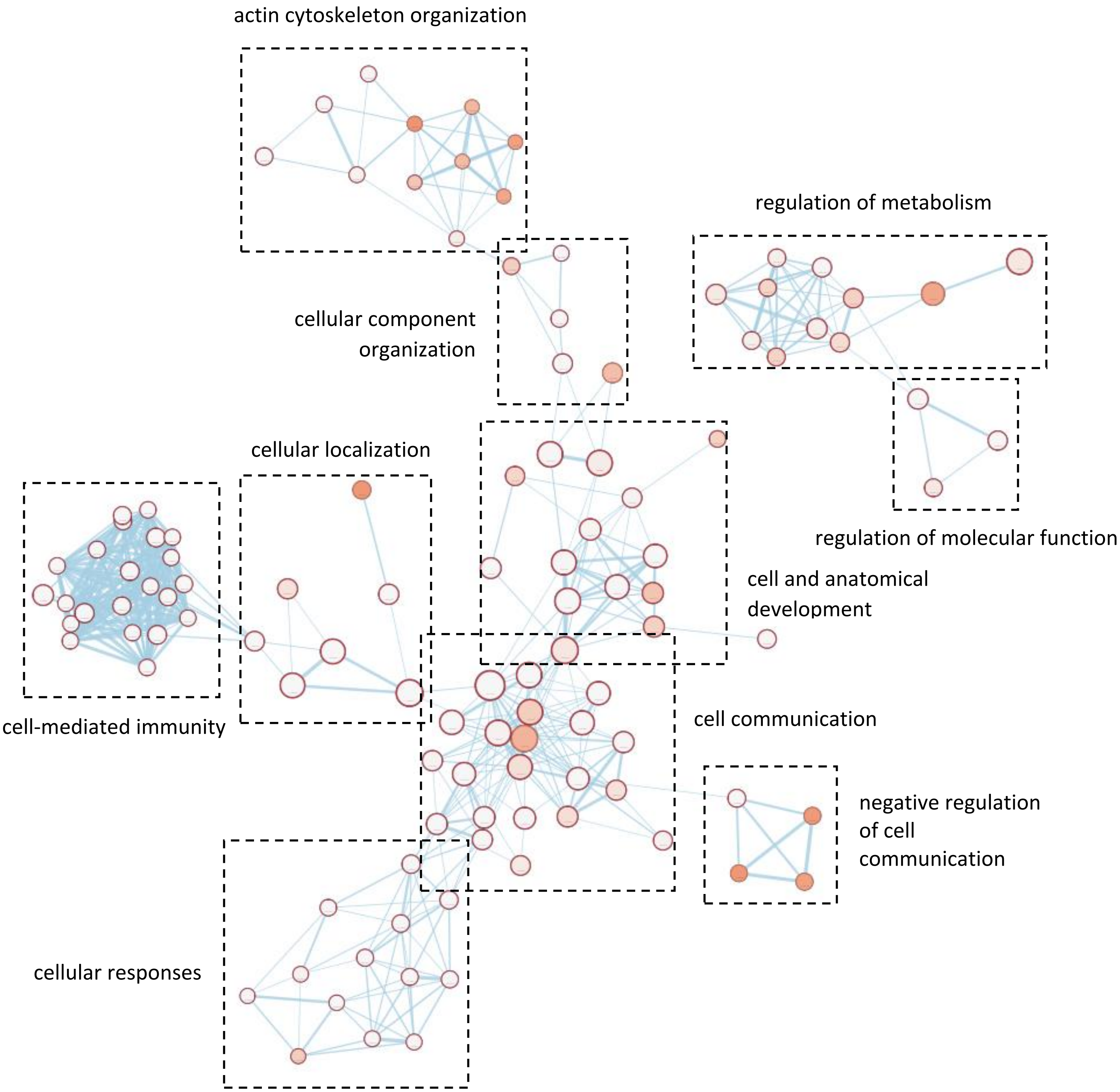

Supplemental Digital Content 6: Enrichment map of the biological processes related to the top 500 genes identified by ANOVA included in the disease non-specific multiclass classifier. The circles are referred to as "nodes" and the connectors are "edges". Nodes represent specific biological pathways and node size represents the number of genes in the pathway. Edges connecting adjacent nodes represent overlapping pathways and edge width represents gene overlap size. The node colour represents the enrichment score. Nodes that are white are more significant (q-value close to 0) and nodes that are red are less significant (q-value close to 1). The nodes are clustered using dashed, rectangular boxes into general functional groups.
